# Synchrotron Mössbauer source: trade-off between intensity and linewidth

**DOI:** 10.1107/S1600577522009316

**Published:** 2022-10-20

**Authors:** Sergey Yaroslavtsev, Aleksandr I. Chumakov

**Affiliations:** a ESRF – The European Synchrotron, CS40220, 38043 Grenoble Cedex 9, France; Paul Scherrer Institut, Switzerland

**Keywords:** synchrotron Mossbauer source, iron borate FeBO_3_

## Abstract

A synchrotron Mössbauer source (SMS) enables conventional (energy-domain) Mössbauer spectroscopy at synchrotron radiation facilities. In comparison with radioactive sources, SMS provides a beam of several micrometres in size, permitting studies of extremely small samples. The SMS linewidth can be narrowed at the expense of its intensity by varying the angular position and temperature of the key element of the SMS – an iron borate ^57^FeBO_3_ crystal. Here, in order to optimize the SMS performance, the angular and temperature dependencies of the SMS parameters have been studied and the optimal angular position and temperature of the crystal have been determined for highest intensity at specified source width. The results show that, when accepting broadening of the source width up to ∼6 natural widths, the intensity of the SMS at the European Synchrotron reaches more than 10^5^ γ-quanta s^−1^. In the opposite extreme, the width of the source approaches the natural width with intensity decreasing to about 10^3^ γ-quanta s^−1^. These changes of intensity up to two orders of magnitude take place over a temperature range of about 0.5°C. For all temperature and angular conditions, the instrumental function of the source was derived; we also analyzed the modification of its shape when passing from the ‘low-width’ to ‘high-intensity’ extremes of SMS operation. Finally, we estimated the influence of the temperature instability and mosaicity of the iron borate crystal on the SMS performance.

## Abstract 2

The synchrotron Mössbauer source at the ESRF is optimized to match specific demands of each user experiment, allowing users to either improve energy resolution or increase intensity by an order of magnitude.

## 1. Introduction

A synchrotron Mössbauer source (SMS) enables conventional (energy-domain) Mössbauer spectroscopy at synchrotron radiation facilities (Smirnov *et al.*, 1997 ▸; Mitsui *et al.*, 2007 ▸; Potapkin *et al.*, 2012*a* ▸). Similar to a radioactive Mössbauer source, SMS provides γ-quanta within a linewidth comparable with the natural width of nuclear resonance. Different to a radioactive source, the γ-quanta from SMS are emitted not in a 4π solid angle but in a collimated beam of size ∼1 mm × 1 mm and divergence of ∼10 µrad.

Such a beam can be focused to a spot with a size of about a few micrometres. This is about six orders of magnitude smaller than the typical area of the beam for a radioactive Mössbauer source. Therefore, studies of extremely small samples taking weeks with a radioactive source can be conducted with SMS in several minutes, and annual scientific programs can be completed within a matter of days.

Therefore, the small beam size makes the SMS extremely useful for studies at high pressure (Potapkin *et al.*, 2013 ▸; Kupenko *et al.*, 2019 ▸; Hamada *et al.*, 2021 ▸), investigations of small iron-bearing inclusions in rocks (Andrault *et al.*, 2018 ▸) and diamonds (Nestola *et al.*, 2016 ▸), meteorites (Blukis *et al.*, 2017 ▸), in surface studies (Cini *et al.*, 2018 ▸; Fujiwara *et al.*, 2021 ▸; Mitsui *et al.*, 2020 ▸), and other scientific cases related to extremely small samples.

Another advantage of SMS is normally the absolute absence of background: by the nature of pure-nuclear diffraction, SMS provides only 14.412 keV radiation, and only its resonant component, behaving as a source with entirely recoilless emission. One more distinction is almost pure (∼98%) linear polarization of the SMS beam.

At the time of writing, SMS is available at ESRF (Smirnov *et al.*, 1997 ▸; Potapkin *et al.*, 2012*a* ▸), SPring-8 (Mitsui *et al.*, 2007 ▸) and PETRA-III (Sergeev, 2021 ▸). It is also under development at some other synchrotron radiation facilities.

In the case of a radioactive Mössbauer source, the source intensity and linewidth are not variable and are predefined by the source. For the SMS case, they are not, but depend on angular position and temperature of the key element of SMS – an iron borate ^57^FeBO_3_ crystal. It is known that increasing the crystal temperature and turning the crystal to a higher angular position provides a narrower source width at the expense of decreased intensity (Smirnov *et al.*, 2011 ▸). What is not known is a way to reach the optimal performance of SMS, *i.e.* to obtain the highest intensity at specified width.

This is what has been investigated in the present work. We studied the angular and temperature dependencies of SMS parameters and determined the optimal angular position and temperature of the crystal for highest intensity at specified source width. We showed that, in order to keep the highest possible intensity when choosing the width, one has to vary both angular position and temperature of the crystal, and we provided a quantitative description for choosing these parameters.

The paper is organized as follows. In Section 2 we describe the experimental setup; Section 3 describes experimental data and the derived SMS parameters; Section 4 reports on optimal conditions for SMS operation; Section 5 discusses instrumental functions; in Section 6 we estimate the influence of temperature instability and slope error of the crystal surface to the SMS parameters; and Section 7 provides conclusions.

## 2. Experimental setup

The study was carried out using the synchrotron Mössbauer source (Potapkin *et al.*, 2012*a* ▸) installed at the Nuclear Resonance beamline (Rüffer & Chumakov, 1996 ▸) ID18 of the European Synchrotron (ESRF). The storage ring was operated in 7/8+1 mode with a mean current of 196 mA.

**Figure 1:**
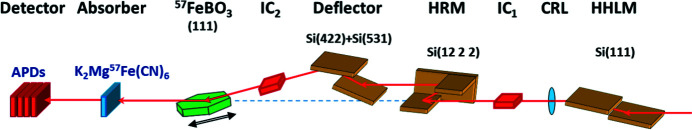
Setup of the experiment. The following abbreviations are used: HHLM – high-heat-load monochromator; CRL – compound refractive lenses; HRM – high-resolution monochromator; APDs – avalance photodiode detectors; IC_1_ and IC_2_ – ionization chambers. The blue dashed line indicates the path of the beam from the HHLM, emphasizing the ‘in-line’ feature of the SMS.

The setup is shown in Fig. 1 ▸. The X-ray beam is prepared using three undulators with magnetic period of 20 mm, gap of 11 mm, total length of 4.8 m, and 14.412 keV radiation component in the fundamental. The high-heat-load monochromator (HHLM) (Chumakov *et al.*, 2014 ▸) decreases the energy bandwidth to 2.0 eV providing 1.0 × 10^14^ γ-quanta s^−1^. The 12 µrad vertical divergence of the beam is collimated down to ∼2 µrad, by one-dimensional parabolic compound refractive lenses (CRLs). In order to decrease the heat load and thereby avoid a temperature gradient on the iron borate crystal, the energy bandwidth is further reduced to 12 meV by a high-resolution monochromator (HRM) – an asymmetrically cut Si(12 2 2) channel-cut crystal.

The deflector is composed of Si(422) and Si(531) crystals and inclines the beam in the vertical plane. The difference in the Bragg angles of the Si(422) and Si(531) reflections matches the Bragg angle of the (111) reflection for the iron borate crystal^1^ with an accuracy better than 100 µrad (Potapkin *et al.*, 2012*a* ▸). Therefore, the beam from the iron borate crystal is emitted practically in the horizontal direction. An adjustment of the tilt angle of the iron borate crystal and distance between this crystal and the deflector allows for nearly exact matching of the SMS beam with the incident beam from the HHLM, *i.e.* providing useful ‘in-line’ geometry of the SMS instrument. This greatly facilitates adjusting the downstream optics and sample environment for user experiments. The beam intensity after the deflector is about 2.0 × 10^11^ γ-quanta s^−1^.

The key element of the SMS is the high-quality ^57^FeBO_3_ iron borate crystal (Kotrbová *et al.*, 1985 ▸) with the residual mosaicity of the crystalline lattice of about 5 µrad (FWHM). The iron borate crystal decreases the energy width of the reflected beam to a bandwidth of about the natural width of the nuclear resonance using the (111) pure-nuclear reflection. This final filtering from a broad synchrotron radiation spectrum of a single-line component with a width of several neV is achieved under specific conditions of pure-nuclear resonance diffraction in an antiferromagnetic crystal in an external magnetic field with combined magnetic and quadrupole hyperfine interaction near the Néel temperature (see Smirnov *et al.*, 1969 ▸, 1986 ▸, 2011 ▸; Smirnov, 2000 ▸, and references therein). Pure-nuclear reflection of the iron borate crystal provides practically ideal suppression of electronic scattering, by at least 12 orders of magnitude as measured for the described setup. Therefore, time spectra of SMS radiation do not contain a prompt peak of electronic scattering (Potapkin *et al.*, 2012*a* ▸). This makes time gating of prompt events unnecessary and allows for using SMS in any mode of storage ring operation.

Similar to a conventional radioactive Mössbauer source, the iron borate crystal is mounted on a standard Mössbauer transducer allowing for modulating the energy of the reflected beam by the Doppler effect. The transducer was operated in a sinusoidal mode in order to avoid high acceleration which can lead to slippage of the gently mounted ^57^FeBO_3_ crystal.

Operation of the SMS requires precise control of the angular position of the iron borate crystal around the Bragg angle of the (111) reflection (θ_B_ = 5.108°), and the temperature of the crystal to be slightly above the Néel temperature (*T*_N_ = 75.20°C). The corresponding accuracies are about 3 µrad and 0.005°C for the angular and temperature control, respectively. An external magnetic field provides a single-magnetic domain state of the crystal with required alignment of the magnetic moments, and smooths the magnetic transition slightly above *T*_N_, making it easier to control the hyperfine magnetic field by temperature. In our setup, an external magnetic field of ∼110 Oe is applied. More details about the experimental setup can be found in Potapkin *et al.* (2012*a* ▸).

The spectrum width and central shift of radiation from the SMS is measured using a standard single-line Mössbauer absorber, K_2_Mg^57^Fe(CN)_6_, with nominal area density of the ^57^Fe isotope of 0.5 mg cm^−2^. For compatibility of the results with the beamline requirements, the absorber was chosen to be identical to one permanently installed at the beamline for express control of SMS instrumental functions. The effective Mössbauer thickness *T* [for definition, see, for example, Smirnov (2000 ▸)] of this absorber is essentially large (∼5) and the intrinsic spectrum^2^ is relatively broad – its width is about 2.1Γ_0_, where Γ_0_ = 0.097 mm s^−1^ = 4.7 neV. On the other hand, such an absorber allows for extremely fast control of the SMS parameters (by checking the spectrum width and central shift), typically in 100 s, which is important for user experiments.

The intensity of the beam after the absorber was monitored by a stack of avalanche photodiode (APD) detectors (Baron, 2000 ▸) with a total efficiency of 86%. In SMS applications, the use of APDs is demanded not by their good time resolution but by their large dynamical range. Relative to photo-multiplier detectors, APDs sustain much higher intensities. This is useful when a strong Renninger (Umweg) reflection of the iron borate crystal is used for alignment of the focusing optics and samples (Potapkin *et al.*, 2012*a* ▸). The detector and absorbers were located at a distance of about 5 m from the iron borate crystal, in the next hutch. Therefore, the background of incoherent scattering was negligible (∼0.5%) and is neglected in data processing.

## 3. Results

We have measured rocking curves – dependencies of the intensity of the beam reflected by the iron borate crystal on the angle of incidence – for various temperatures of the crystal slightly above the Néel temperature, and Mössbauer spectra of the K_2_Mg^57^Fe(CN)_6_ absorber for various angular positions and temperatures. Before each measurement of the rocking curve, the optics was re-adjusted for accurate determination of the beam intensity at various angular positions for a given temperature.

### 3.1. Rocking curves and Mössbauer spectra

At room temperature, the rocking curve of the ^57^FeBO_3_ iron borate crystal is close to Lorentzian shape with a width of about 20 µrad (FWHM). This is slightly larger than the 15 µrad expected from theory. The difference gives a rough estimate of the slope error of the crystal surface of ∼5 µrad. The same value of the slope error was obtained in analysis of the vertical spot size of the focused SMS beam.

**Figure 2:**
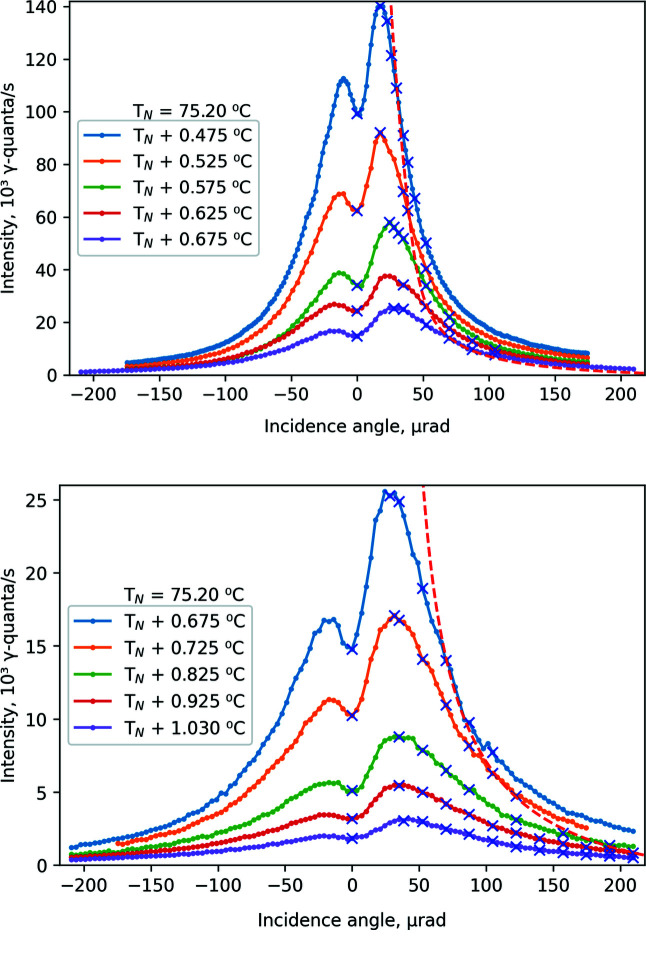
The evolution of rocking curves with temperature for lowest (top panel) and highest (bottom panel) temperatures. The incidence angle θ is given as the deviation from the Bragg angle determined by accounting for the shift due to refraction of the incident beam at the entrance into the crystal. Blue crosses indicate the angular positions where the Mössbauer spectra were measured. The red dashed line shows the optimal conditions for SMS operation (see text).

Starting from *T*_N_, the shape of the rocking curve changes quickly. Fig. 2 ▸ shows the evolution of the rocking curve with temperature for elevation Δ*T* = *T* − *T*_N_ above the Néel temperature by Δ*T* = 0.5–1°C. The rocking curve acquires an asymmetric, double-peak shape, with lower left and higher right peaks. This shape is defined by nearly collapsed hyperfine structure of nuclear levels consisting of two resonances with close energies but different amplitudes of scattering (Smirnov *et al.*, 1969 ▸, 1986 ▸, 2011 ▸; Smirnov, 2000 ▸). The exact Bragg position corresponds to the local minimum between the peaks (Smirnov *et al.*, 2011 ▸).

The Mössbauer spectra were measured for zero and positive values of angle θ. Blue crosses in Fig. 2 ▸ indicate points where spectra were collected. Measurements at negative angles were not performed, because at negative θ instrumental functions have pronounced satellites (Smirnov *et al.*, 2011 ▸; Potapkin *et al.*, 2012*b* ▸) and intensity is obviously less.

**Figure 3:**
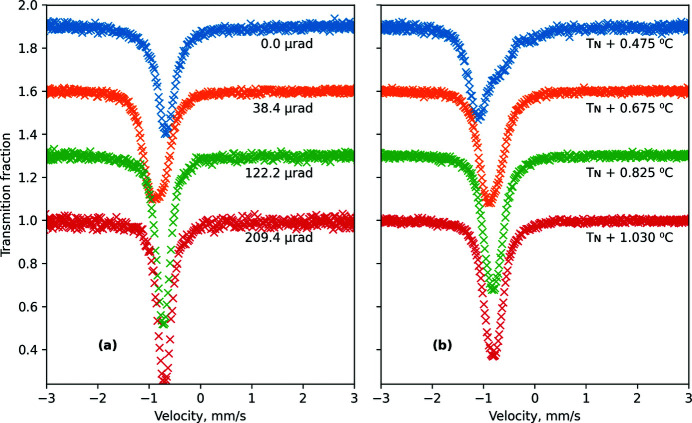
Mössbauer spectra of the standard K_2_Mg^57^Fe(CN)_6_ absorber collected (*a*) at temperature *T* = 76.230°C (*T*_N_ + 1.03°C) for various angular positions θ and (*b*) at θ = 52 µrad for various temperatures. For better visibility, each subsequent spectrum is shifted vertically by 0.30 (*i.e.* by 30% of transmission).

The velocity scale (channel price) was calibrated using an α-iron foil with natural abundance of the ^57^Fe isotope and thickness of 25 µm. In Fig. 3 ▸ the Mössbauer spectra are shown raw but folded, and the velocity scale corresponds to the transducer velocity.

In this experiment, we change the source properties by changing the SMS conditions while the absorber remained the same. This meant that changes of the position of the transmission minimum are due to a change in the source but not the sample. For example, the shift of the observed spectrum line to a positive direction actually means a corresponding shift of the source line to the negative direction, and vice versa.

The variations of the spectra with temperature and incidence angles are shown in Fig. 3 ▸. At the peak of the rocking curve the spectrum becomes rather broad (see, for example, Fig. 3 ▸, the spectrum measured at an incidence angle of 38 µrad) but intensity reaches the maximum possible at this specific temperature. Increasing the incidence angle leads to a narrower spectrum, lower intensity and a shift of the spectrum line in the positive direction. Increasing temperature provides the same changes [Fig. 3 ▸(*b*)].

### 3.2. Derived parameters of SMS

**Figure 4:**
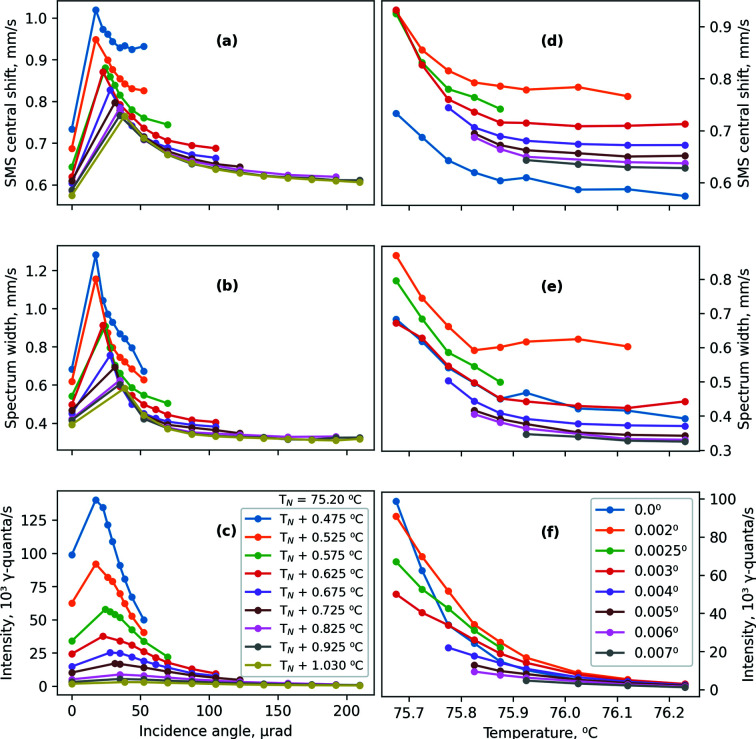
Summary of the derived SMS parameters. The SMS central shift (*a*, *d*), spectrum width (*b*, *e*), and intensity (*c*, *f*) are shown for various temperatures as a function of incidence angle (*a*–*c*) and for various angles as a function of temperature (*d*–*f*).

The derived parameters of the central shift, spectrum width and intensity of the SMS are shown in Fig. 4 ▸ for various temperatures and angular positions of the iron borate crystal. Obviously, in order to keep a source in resonance with a hypothetical absorber with zero central shift, a central shift of the source to, for example, positive velocity would require moving the source with opposite, that is, negative velocity. Therefore, the central shifts of the SMS are obtained as the central shift of the standard K_2_Mg^57^Fe(CN)_6_ absorber (−0.097 mm s^−1^, relative to α-iron) minus the central shifts of the measured Mössbauer spectra.^3^ The spectrum widths of the SMS are given as the spectrum widths of the measured Mössbauer spectra. The intensity values are given as observed in the measurements of the rocking curves (Fig. 2 ▸), without corrections for detector efficiency. The central shift and spectrum width show stronger angular and temperature dependencies in the small-angle and lowest-temperature regions. At higher angles and temperatures, the dependencies becomes less pronounced, resembling an approach to asymptotic values. This behavior has important practical implications on stability and quality of SMS operation (discussed below).

## 4. Optimal operation

### 4.1. Intensity

**Figure 5:**
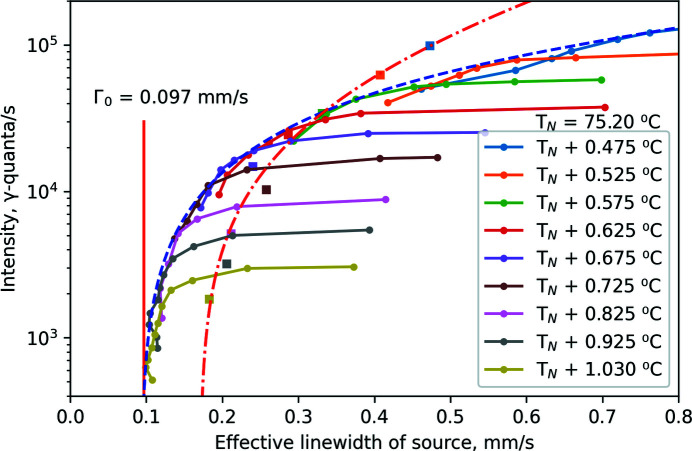
The master plot of all experimental data in the width–intensity representation. The solid lines connect data points (circles) measured at fixed temperatures for various angles. The displacement of solid lines from the bottom-left to top-right corners corresponds to decreasing of the angular position θ. The envelope (blue dashed line) shows the maximum available intensity at specified effective width of the source. The squares connected by the red dash-dot line show the data points measured for θ = 0 angular position at indicated temperatures. The red solid vertical line shows the natural width Γ_0_.

For many applications, optimal operation of SMS corresponds to maximum intensity at a specified linewidth of the source. These conditions can be determined using Fig. 5 ▸, where intensity as a function of linewidth is plotted for all data measured at various temperatures and angles. Data points obtained at fixed temperatures for various angles are connected by solid lines. At each specific temperature, intensity decreases with increasing angle. Data measured at angular position θ = 0 for various temperatures are shown separately, connected by the red dash-dot line.

The highest intensity for any specified linewidth is described by the upper envelope (assumed to be smooth and monotonic) of the presented data, shown by the blue dashed line in Fig. 5 ▸. For the largest linewidths, higher intensity can be obtained at angle θ = 0 but, as discussed below, this option requires extremely high accuracy of angular positioning and crystal quality.

Exact determination of the source linewidth requires special procedures, as discussed below. Therefore, data in Fig. 5 ▸ are reported in terms of the ‘effective linewidth’ of the source, which is calculated as the difference between the linewidth of the measured Mössbauer spectrum [as shown in Figs. 4 ▸(*b*) and 4(*e*)] and the intrinsic linewidth^4^ (0.21 mm s^−1^) of the utilized single-line absorber. As discussed below, the effective linewidth is not exactly equal to the linewidth of the instrumental function. However, it gives a useful means to estimate the linewidth of the expected Mössbauer spectrum for other samples, by adding the effective linewidth of the source to the intrinsic linewidths of the samples.

**Figure 6:**
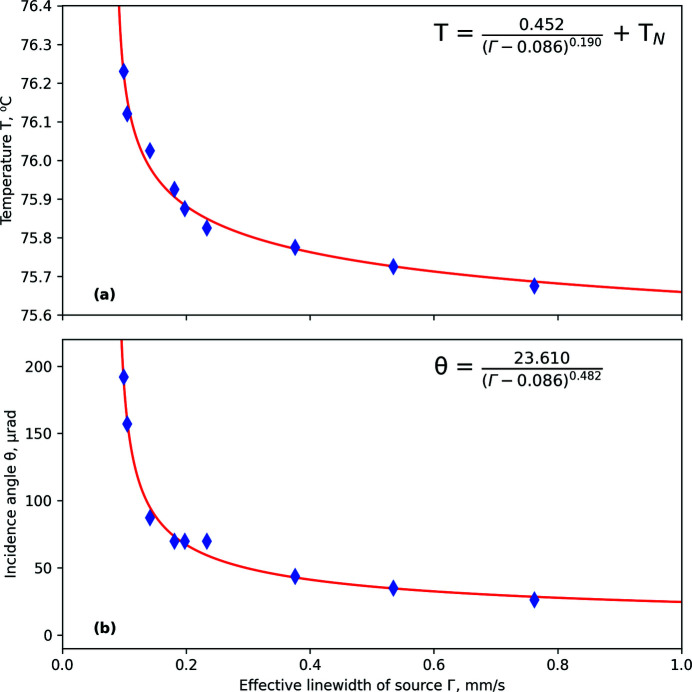
Selection of experimental data points closest to the envelope (blue dashed line in Fig. 5 ▸) for each studied temperature in the (*a*) width–temperature and (*b*) width–incidence angle representations. The red solid lines are the fits through the optimal points, shown by the equations at the top of each panel.

Following the ‘optimal operation’ blue-dash envelope in Fig. 5 ▸ from top-right to bottom-left reveals that keeping the highest intensity while choosing a smaller linewidth requires a simultaneous increase of both angle and temperature. The way to choose the angular position and temperatures corresponding to optimal operation with a given linewidth is reported in Figs. 6 ▸(*a*) and 6(*b*). Here temperatures [Fig. 6 ▸(*a*)] and angles [Fig. 6 ▸(*b*)] are shown as a function of linewidth for selected data points, where the observed intensity is most close to the ‘optimal operation’ envelope shown in Fig. 5 ▸ by the blue dashed line.

In a first approximation, the values of angles and temperatures required for optimal operation with specified linewidth are given by the presented data points. The fit is shown by red solid lines and reported by the numerical expressions shown at the top of Figs. 6 ▸(*a*) and 6(*b*). The expressions are not theoretical but heuristic ones – they are chosen as a simplest way to approximate data by dependencies with both vertical and horizontal asymptotes. The vertical asymptote represents the lower limit of the effective linewidth of the source, whose existence is theoretically expected (Smirnov *et al.*, 2011 ▸). The horizontal asymptote for incidence angle is chosen as zero, because the largest intensity which corresponds to largest linewidth is observed for rocking curves collapsed at low temperatures to a single peak centered at zero angle (Smirnov *et al.*, 2011 ▸). The horizontal asymptote for temperature is chosen as the Néel temperature because, approaching *T*_N_, the linewidth starts growing rapidly due to coming magnetic splitting of the spectrum.

By fitting the data with the described choice of asymptotes and rational functions shown in Figs. 6 ▸(*a*) and 6(*b*), the asymptotic linewidth was found to be ∼0.086 mm s^−1^ (∼0.9Γ_0_). This gives an indication of the smallest effective linewidth, which can be reached at the highest angles and temperatures with intensity approaching zero. Experimentally, the smallest observed effective linewidth is ∼1Γ_0_, with an intensity of about 10^3^ γ-quanta s^−1^. In most experiments, ESRF users are served with an effective linewidth of about 0.2 mm s^−1^ (∼2Γ_0_) and intensity of about 2 × 10^4^ γ-quanta s^−1^ as a reasonable compromise between the width and intensity.

### 4.2. Signal-to-noise ratio

In the next step of optimization, the optimal operation can be defined as the conditions providing the best data quality, *i.e.* the highest signal-to-noise ratio (SNR) of the measured spectra. In this approach, the effective linewidth cannot be chosen arbitrary, but should be found in a compromise between the signal (magnitude of the absorption dip in the measured spectrum) decreasing with the source linewidth in the upper-right extreme of the data shown in Fig. 5 ▸ and the relative noise (inverse square root of collected counts), increasing with lower intensity at the lower-left extreme of the data.

Assuming that the shape of the spectra does not change significantly with the source linewidth, the SNR can be evaluated as the square root of the intensity divided by the width of the measured spectrum (FWHM) and multiplied by the square root of time 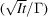 . Obviously, the evolution of the SNR with the effective source linewidth should depend on the intrinsic width of the utilized sample, because increasing the effective linewidth provides stronger broadening in spectra for samples with smaller intrinsic width, and vice versa.

**Figure 7:**
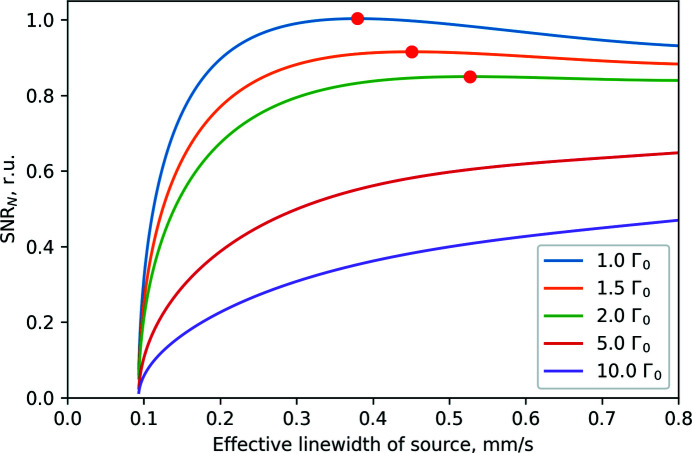
Estimated normalized signal-to-noise ratio (SNR_*N*_) for indicated intrinsic spectrum widths. Estimation was made using the envelope (blue dashed line) from Fig. 5 ▸. Red dots indicate maximums.

Fig. 7 ▸ shows the dependence of the SNR on the source linewidth for several intrinsic widths of the studied samples. The SNR values are calculated for equal measurement times, and normalized to bring the highest evaluated SNR value to unity.

Comparison of Figs. 5 ▸ and 7 ▸ shows that the highest intensity is not necessarily beneficial for good data quality. For samples with small intrinsic linewidths, the SNR decreases at high source linewidths, revealing optimal operation conditions at intermediate intensities and source linewidth, as shown by the red circles. For samples with moderated intrinsic linewidth (<3Γ_0_), the SNR does not increase much at high source linewidth. In the case of very broad intrinsic linewidths (∼10Γ_0_), which could be observed for example for glasses, the SNR increases with source linewidth. Operation at the highest source linewidths, as discussed below, is related to more asymmetric instrumental functions with longer tails and even satellites, but could still be useful.

## 5. Instrumental function

Presently most SMS data are fitted assuming a Lorenz-squared shape of the instrumental function (Prescher *et al.*, 2012 ▸). Although a squared Lorenz is a good first approximation, it is not the exact shape of the instrumental function (Smirnov *et al.*, 2011 ▸). The best approach would be to determine the instrumental function experimentally, for specific conditions of SMS operation. This is important for data measured with high statistical accuracy, and crucial for those cases where the instrumental function significantly deviates from a squared Lorenz, *i.e.* at low temperatures and incidence angles, chosen for studies requiring the highest intensity.

### 5.1. Determination of the intrinsic spectrum of the standard absorbed and instrumental function of SMS

For effective operation, determination of the instrumental function should take a sufficiently shorter time than measurements of the Mössbauer spectra for the studied samples. For this purpose, the beamline is equipped with a standard single-line Mössbauer absorber K_2_Mg^57^Fe(CN)_6_, the same as used in this study. It is permanently installed in the optics hutch, and measurements of the instrumental function can be made while changing samples in another experimental hutch. Due to large resonance absorption and low photo-absorption, measurements of the Mössbauer spectrum with high statistical accuracy for this absorber do not take a long time.

For reliable determination of the instrumental function from the measured spectrum of the standard absorber, the intrinsic spectrum of the absorber should be known with high accuracy. This is achieved in a separate procedure, using the standard single-line absorber and an additional reference sample of α-iron foil (natural-enrichment, thickness of 25 µm). First, the Mössbauer spectrum of the reference α-iron sample is measured with a conventional radioactive Mössbauer source. Then, the Mössbauer spectra of both reference α-iron sample and single-line absorber are measured with SMS, assuring that the instrumental function of SMS stays the same for both measurements.

Simultaneous fitting of α-iron spectra measured with the conventional source and with SMS allows one to extract the intrinsic spectrum of α-iron (the same for both measurements) and the instrumental function of SMS. Here the instrumental function of a radioactive source is assumed to have Lorentzian shape with variable width. Magnetic splitting of the spectrum to six lines with different resonance cross sections helps to distinguish the effects of line broadening caused by thickness and by inhomogeneous distribution of hyperfine parameters. Known values of the hyperfine magnetic field, Lamb–Mössbauer factor and thickness also facilitate the fit. Finally, when the instrumental function of the SMS is determined from α-iron data, it is applied to fit the measured spectrum of the single-line absorber, providing its intrinsic spectrum.

The described procedure is somewhat time-consuming, but it has to be done once, and only for a single temperature and angular parameters of the SMS. Afterwards, when the intrinsic spectrum of the standard single-line absorber is determined, the instrumental function for any conditions of SMS operation can be found by fast measurements of the standard absorber spectrum. The procedures described above are built into a single software package for fitting SMS spectra. More details of the fitting routine will be provided elsewhere (Yaroslavtsev, 2022 ▸).

### 5.2. Discussion of the instrumental functions

**Figure 8:**
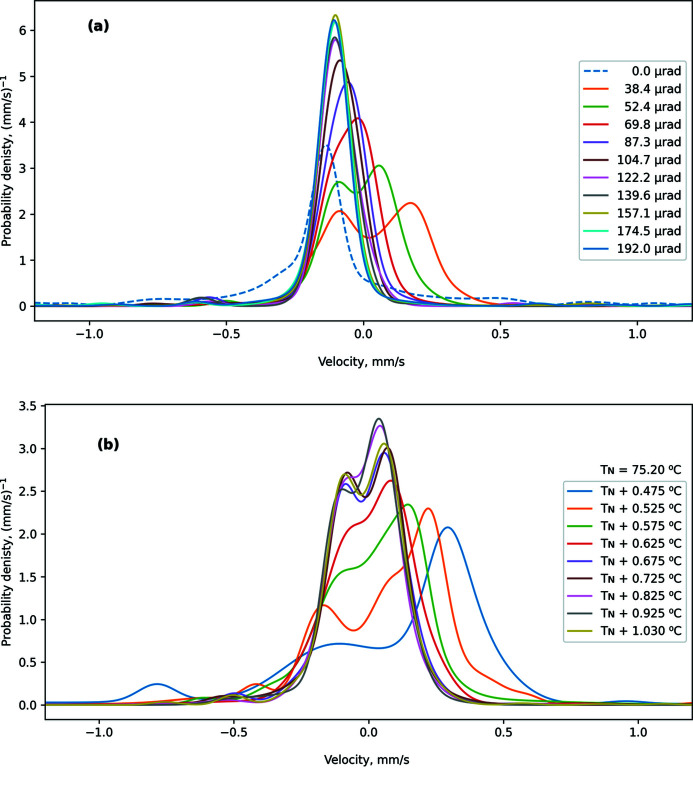
Instrumental functions for various angular positions at temperature *T* = 76.230°C (*a*) and for various temperatures at the incidence angle θ = 52 µrad (*b*).

Examples of instrumental functions reconstructed from spectra of a standard single-line absorber are illustrated in Fig. 8 ▸. The area of each instrumental function is equal to unity. Increasing incidence angle leads to a narrower instrumental function [Fig. 8 ▸(*a*)]. The same effect is provided by increasing temperature [Fig. 8 ▸(*b*)]. For narrow instrumental functions (low-intensity regime), the shape is close to the theoretically expected (Smirnov *et al.*, 2011 ▸) squared Lorentzian curve. At nominal conditions for operation of SMS at ESRF (with an effective width of about 0.2 mm s^−1^, at *T* = *T*_N_ + 0.625°C and θ = 70 µrad), the instrumental function is still a single line.

At low angles and low temperatures (high-intensity regime), the shape of the instrumental function changes significantly. It acquires a double-peak shape, becomes strongly asymmetric, and shows additional satellites at lower energies. This evolution is caused mainly by interference of purely resolved hyperfine nuclear transitions in the iron borate crystal in the vicinity of the Néel temperature (Smirnov *et al.*, 2011 ▸). Additional broadening and asymmetry comes from uncertainties in incidence angle (caused by mosaicity of the iron borate crystal) and temperature, as discussed below. Due to these uncertainties, the actual instrumental function is a superposition of theoretically expected instrumental functions with various central shifts and widths, and with some correlation between these parameters (see Fig. 4 ▸).

When an experiment aims to evaluate small impurities relative to the main dominating components or in studies of purely resolved spectra, an obvious choice is operation with a narrow instrumental function at the expense of intensity. In the opposite regime of large linewidths, the complicated shape of the instrumental function will lead to blurring of small resonance lines by tails of bigger ones. However, fitting data with a reliable instrumental function can fix these issues, at least partly (Yaroslavtsev, 2022 ▸). Thus, the high-intensity regime with broad, asymmetric and complicated instrumental functions can still be useful in those cases where spectra should be collected for a very short time (a few minutes), for example in the case of *operando* experiments, or for measurement of broad Mössbauer spectra, for example for glasses (Sinmyo *et al.*, 2019 ▸; Dorfman *et al.*, 2020 ▸), and in Rayleigh scattering of Mössbauer radiation (Mitsui *et al.*, 2022*a* ▸,*b* ▸).

Finally, we note that, due to the complicated shape of the instrumental function, the width of the measured spectrum is not a sum of the width of the intrinsic spectrum and the width of the instrumental function, even for ideally thin and homogeneous samples. Most significant differences appear for narrow instrumental functions. Numerical calculations show that if, for example, the width of the instrumental function is 0.13 mm s^−1^ (close to the narrowest achieved in this study) and the intrinsic spectrum of the sample has a Lorentzian shape with the same width, the experimental spectrum will have a width of 0.23 mm s^−1^, not 0.26 mm s^−1^. Thus, the effective width (difference between the widths of measured and intrinsic spectra) is smaller than the real width of the instrumental function, at least when the instrumental function is still a single line.

## 6. Effect of accuracy of temperature control and quality of iron borate crystal on SMS performance

As discussed above, the central shift and linewidth of SMS radiation depend on the temperature and angular position of the iron borate crystal. Therefore, the temperature distribution on the crystal, finite accuracy of temperature control, and incident angle distribution caused by finite mosaicity (slope error) of the crystal may cause broadening of the SMS linewidth. In this section, we analyze the influence of the temperature instability and slope error of the iron borate crystal on the SMS performance.

In terms of temperature distribution, the first factor to consider is the finite accuracy of temperature control, of about ±0.005°C in this study.^5^ The uncertainty is apparently determined by the thermal stability of the furnace and the quality of the temperature controller (Lake Shore 332); it does not change during SMS operation. The second factor is inhomogeneity of the external magnetic field on the borate crystal of about ±4 Oe. Its influence on SMS performance is equivalent to a temperature inhomogeneity of about ±0.012°C (Potapkin *et al.*, 2012*a* ▸). In terms of angular uncertainties, one factor is the finite accuracy of angular positioning, of about 3 µrad. This value is determined mainly by the accuracy of the angular positioning of the rotation stage (Huber two-circle segments 5203.80). In user experiments, the correct angular position is permanently controlled by monitoring the isomer shift of a standard single-line absorber, normally before and after each sample measurement. Corrections of the angular position are required typically a few times per week, if any. Another factor is the mosaicity of the crystal, of about 5 µrad.

The variations of the central shift and effective linewidth caused by the mentioned temperature and angular uncertainties can be estimated from the slopes of the temperature and angular dependencies of the central shift and spectrum linewidth shown in Fig. 4 ▸. In Table 1 ▸, these estimations are reported for nominal conditions of the SMS operation at ESRF, with effective linewidth of about 0.2 mm s^−1^, corresponding to *T* = 75.825°C (*T*_N_ + 0.625°C) and incidence angle of 0.004° (70 µrad). In addition, Table 1 ▸ shows variations of the central shift and effective linewidth of Mössbauer spectra measured using different parts of the iron borate crystal, selected by the downstream slit in front of the APD detector.

Experimentally measured variations match the estimations with reasonable accuracy. The estimations in Table 1 ▸ show that the most affected parameter is the effective linewidth, not the central shift; and that the main effect on both parameters is caused by crystal mosaicity. Therefore, the possible temperature gradient over the crystal surface does not seem to be significant. Overall, in nominal operation, the variations of the central shift and effective linewidth are essentially small, specifically less than 5% and 8% of the effective linewidth, respectively.

For operation with smaller effective linewidths, corresponding to higher temperatures and angular positions, the slope of the temperature and angular dependencies of the central shift and linewidth is less pronounced (Fig. 4 ▸). Therefore, in these cases the influence of temperature and angular uncertainties on SMS performance is expected to be smaller. On the contrary, for operation with larger linewidths, related to smaller temperatures and angles, the central shift and linewidth have a steeper slope (Fig. 4 ▸). Therefore, operation of SMS with large effective linewidth will be more strongly affected by the crystal mosaicity and the finite accuracy of temperature and angular control. In particular, near the angular position θ = 0, the angular dependencies of the central shift and linewidth are extremely strong (Fig. 4 ▸). Thus, operating in this position requires an iron borate crystal with very small mosaicity, and needs very high accuracy of angular positioning.

In addition to the above-considered ‘spectroscopic’ aspects of SMS operation, the mosaicity of the iron borate crystal is directly related to the ability to focus SMS radiation. In the approximation of the SMS and focusing mirror located far away from the source and relatively close to each other, the mosaicity α of the crystal causes an increase of the vertical beam size of the focused beam by about 2α*f*_0_, where *f*_0_ is the focal distance. For the mentioned mosaicity of the iron borate crystal (5 µrad) and the focal distance of the presently used focusing mirror (∼1 m), this limits the vertical beam size to 10 µm, which is close to the typical vertical size of the focused SMS radiation at ESRF (∼12 µm × 4 µm, vertical  ×  horizontal).

## 7. Conclusions

We established the protocol of finding the optimal parameters for operating a synchrotron Mössbauer source in order to reach the maximum available intensity at a specified source width. The optimization involves adjustment of both angular position and temperature of the iron borate crystal. In all cases, a narrower width can be obtained at the expense of decreasing intensity. When accepting broadening of the width up to ∼6 natural widths, the intensity of the SMS at the European Synchrotron reaches more than 10^5^ γ-quanta s^−1^. In the opposite extreme, the width of the source approaches the natural width with intensity decreasing to about 10^3^ γ-quanta s^−1^.

We also developed the protocol of accurate and fast determination of the instrumental function of the source and analyzed the instrumental functions at various angular positions and temperatures. In the narrowest width limit, in accordance with theory, the shape of the instrumental function is close to a squared Lorentzian curve. A shift of the operation parameters to the broader width but higher intensity regime makes the instrumental function asymmetric, but it still keeps approximately a single peak shape up to a width of ∼3Γ_0_. Further, gaining intensity at the expense of the source width qualitatively changes the shape of the instrumental function to a double or triple peak profile. However, even under these conditions it can be used for successful data processing, in experiments where high intensity is required for express measurements or where an intrinsic spectrum is extremely broad, for examples for glasses or for Rayleigh scattering of Mössbauer radiation.

Finally, we analyzed the influence of the slope error (mosaicity) of the iron borate crystal, the accuracy of angular positioning and temperature control, and other related parameters on the SMS performance. The estimation shows that deviations of these parameters from ideal quality do not influence much the ‘spectroscopic’ aspects of the SMS performance, providing only a negligible broadening of the SMS linewidth. However, the slope error (mosaicity) of the iron borate crystal has a crucial impact on focusing of SMS radiation, limiting the vertical size of focused beam.

## Figures and Tables

**Table 1:** Estimated variations of the central shift (CS) and effective linewidth (LW) of SMS radiation caused by various contributions, and as measured for different parts of the iron borate crystal

Contribution	CS (mm s^−1^)	LW (mm s^−1^)
Accuracy of temperature control (±0.005°C)	±0.001	±0.003
Inhomogeneity of magnetic field (±4 Oe)	±0.002	±0.006
Accuracy of angular positioning (3 µrad)	±0.004	±0.007
Mosaicity of crystal (5 µrad)	±0.007	±0.012
Measured for different parts of crystal	±0.009	±0.013

## Footnotes

1 For the iron borate crystal, indexes are given in the rhombohedral coordinate system.

2 The hypothetical spectrum measured with the instrumental function (source energy distribution) equal to the delta-function.

3 Here the central shift of the Mössbauer spectrum is defined as the middle of two velocities at half minimum of transmission. The spectrum width of the Mössbauer spectrum is defined as the full width at half-minimum.

4 Here, ‘intrinsic width’ means the linewidth of a Mössbauer spectrum which would be measured for a given sample using a source whose energy distribution has the shape of a Dirac delta function.

5 At present, the accuracy of temperature control is improved to ±0.001°C.
